# P-2005. Evaluating a Semiquantitative Multiplex PCR for Diagnosis of Bloodstream Infections: Real-World Data from a North Indian Tertiary Care Centre

**DOI:** 10.1093/ofid/ofaf695.2169

**Published:** 2026-01-11

**Authors:** Sandeep Rao Kordcal, Md Tariq Maula, Bharathi Arunan, Vishal Keerthy Kumar, Animesh Ray, Sarita Mohapatra, Siddharth Jain, Manish Soneja, Naveet Wig

**Affiliations:** All India Institute of Medical Sciences, New Delhi, Delhi, India; All India Institute of Medical Sciences, New Delhi, Delhi, India; All India Institute of medical Sciences, New Delhi, new delhi, Delhi, India; All India Institute of Medical Sciences, New Delhi, Delhi, Delhi, India; All India Institute of Medical Sciences, New Delhi, Delhi, Delhi, India; All India Institute of Medical Sciences, New Delhi, Delhi, Delhi, India; All India Institute of Medical Sciences, New Delhi, Delhi, Delhi, India; All India Institute of Medical Sciences, New Delhi, Delhi, India; All India Institute of Medical Sciences, New Delhi, Delhi, Delhi, India

## Abstract

**Background:**

Bloodstream infections (BSIs) are a major contributor to mortality, necessitating prompt and accurate pathogen identification to guide effective antimicrobial therapy. The BioFire Blood Culture Identification 2 Panel (BF BCID 2P), a multiplex PCR based assay, promises rapid pathogen detection and resistance marker identification with a turnaround time of approximately one hour. This study evaluates the diagnostic utility of the BCID panel compared to the standard-of-care (SoC) in patients with suspected BSIs.

**Figure:**
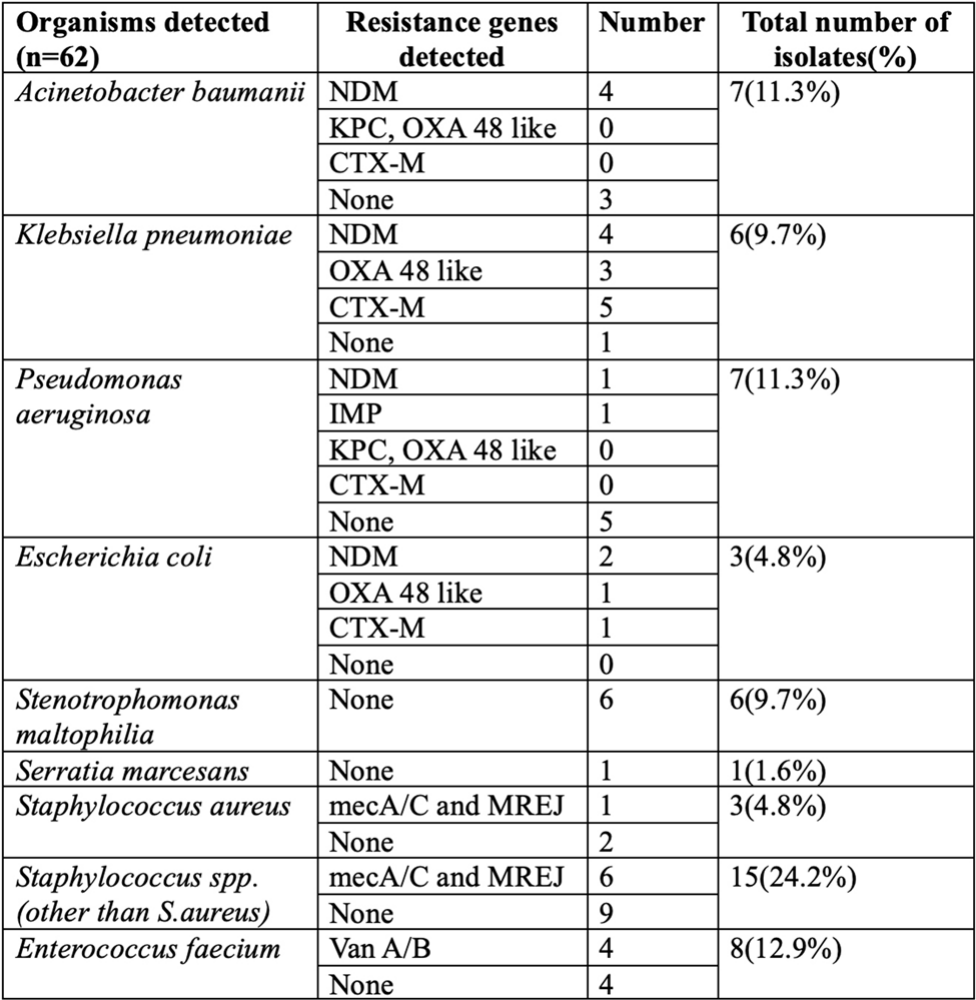
Organisms and anti-microbial resistance genes detected by multiplex PCR in patients with suspected bloodstream infection

**Figure:**
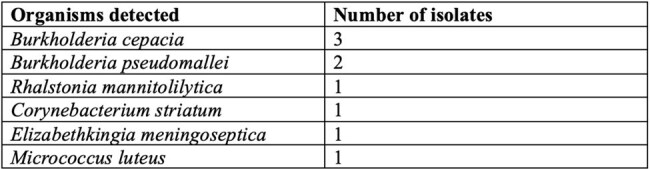
Organisms isolated by standard-of-care with targets not available on multiplex PCR

**Methods:**

This retrospective observational study included data from patients managed at our tertiary care hospital between August 2023 and December 2024. Adult patients with positive blood cultures (PBCs) identified by an automated continuous monitoring blood culture system who were tested using the BCID panel within 24 h of positivity were included in the study. Standard-of-care (SoC), defined as subculture and isolate identification of PBCs was used as the comparator. Positive and negative predictive agreement and average lead time between the two tests were measured.

**Figure:**
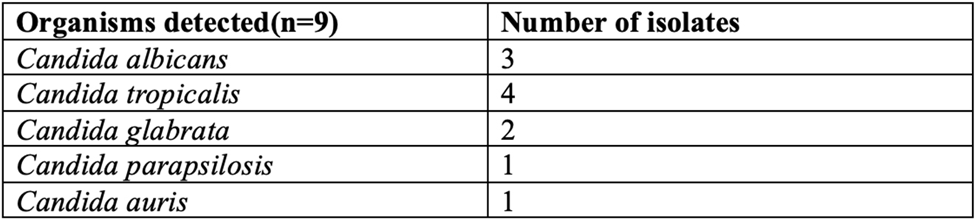
Fungal pathogens identified by multiplex PCR in patients with suspected bloodstream infection

**Results:**

A total of 74 patients were included, with 79 samples analysed. BCID identified bacterial pathogens in 62(78.5%), of samples compared to 57(72.2%) by SoC. The average lead time of BCID over SoC was 70.15 hours (range: 14.53 to 139.87 hours). PPA and NPA for BCID were 88.1% and 68.4% respectively. BCID detected fungal pathogens in 9(11.4%), of cases. Out of the clinical records available(n=50), there was potential for antimicrobial therapy adjustments guided by BCID reports in 45(90%) cases, with potential for appropriate escalation in 34(78%) and appropriate de-escalation in 7(14%). The prevalence of metallo-β-lactamase producers was notably high at 40.6% (13/32), while methicillin resistance among *Staphylococcus* isolates was observed in 38.89% (7/18) of cases.

**Conclusion:**

BioFire BCID 2 Panel demonstrates substantial utility in real-world antimicrobial stewardship with a significant lead time of approximately 70 hours over conventional blood cultures and strong positive predictive agreement. However, its inability to detect pathogens outside its target spectrum highlights the ongoing necessity of conventional blood culture techniques.

**Disclosures:**

All Authors: No reported disclosures

